# Intracranial Metastatic Neuroblastoma Treated with Gamma Knife Stereotactic Radiosurgery: Report of Two Novel Cases

**DOI:** 10.1155/2012/690548

**Published:** 2012-01-19

**Authors:** Nathan C. Rowland, Jennifer Andrews, Daxa Patel, David V. LaBorde, Adam Nowlan, Bradley George, Claire Mazewski, Andrew Reisner, Howard M. Katzenstein

**Affiliations:** ^1^Department of Neurological Surgery, University of California, San Francisco, 505 Parnassus Avenue, Room 779 M, San Francisco, CA 94143-0112, USA; ^2^Aflac Cancer Center, Department of Pediatrics, Children's Healthcare of Atlanta, 1405 Clifton Road NE, Third Floor, Atlanta, GA 30322, USA; ^3^Division of Neurolosurgery, University of Alabama at Birmingham, 510 Twentieth Street South, 1060 Faculty Office Tower, Birmingham, AL 35294-3410, USA; ^4^Department of Neurosurgery, Emory University School of Medicine, 1365 Clifton Road NE, Building B, Suite 2200, Atlanta, GA 30322, USA; ^5^Department of Radiation Oncology, Piedmont Hospital, 1968 Peachtree Road NW, Atlanta, GA 30309, USA; ^6^Pediatric Neurosurgical Associates, Children's Healthcare of Atlanta, 5455 Meridian Mark Road NE, Suite 540, Atlanta, GA 30342-1654, USA

## Abstract

Intracranial metastasis of neuroblastoma (IMN) is associated with poor survival. No curative therapy for the treatment of IMN currently exists. Unfractionated radiotherapy may be beneficial in the treatment of IMN given the known radiosensitivity of neuroblastoma as well as its proclivity to metastasize as discrete lesions. We present two patients with IMN treated with Gamma Knife stereotactic radiosurgery (SRS). Single-fraction radiotherapy yielded temporary reduction of tumor burden and stability of disease in both patients. SRS may be a useful palliative tool in the treatment of IMN and expands the overall treatment options for this disease.

## 1. Introduction

Neuroblastoma is the most prevalent extracranial solid tumor type occurring in childhood [1–3]. Intracranial metastasis of neuroblastoma (IMN), though somewhat rare, reduces median survival to less than 14 months [4, 5]. The radiosensitivity of neuroblastoma is routinely exploited during treatment of primary sites of high-risk disease, persistent metastatic lesions, and recurrent local tumor [4, 6–8]. Gamma Knife stereotactic radiosurgery (SRS) may therefore represent an additional therapeutic option of delivering radiotherapy to intracranial sites while minimizing toxicity to critical surrounding structures. SRS has been previously applied to primary central nervous system (CNS) neuroblastoma [9, 10] and olfactory esthesioneuroblastoma (Table 1) [11]. To our knowledge, SRS has not been applied to recurrent neuroblastoma with CNS metastasis. We report two cases of high-risk disease in which SRS was used to temporarily arrest recurrent IMN.

## 2. Case Reports

### 2.1. Case 1

#### 2.1.1. Initial Diagnosis and Treatment

A 5-year-old male presented to our clinic with a 1-week history of intermittent fevers and abdominal pain. Multiple cervical and axillary lymph nodes were palpable. Subsequent computed tomography (CT) imaging showed a right-sided suprarenal mass, and a ^123^I-metaiodobenzylguanidine (MIBG) scan showed diffuse metastatic disease. Bone marrow biopsy revealed the diagnosis of neuroblastoma without amplification of myelocytomatosis viral-related oncogene or MYCN. The patient was started on induction chemotherapy per Children Oncology Group's (COG) ANBL00P1 protocol, which includes vincristine, cyclophosphamide, and cisplatin, as well as topotecan, doxorubicin, and etoposide [8]. Following chemotherapy, bone marrows became negative, and a complete resection of the primary tumor was performed. The patient then received tandem high-dose chemotherapy and autologous stem cell rescue (HDC/SCR) followed by radiation to the primary tumor site and a 6-month course of oral isotretinoin.

#### 2.1.2. Recurrence, Gamma Knife Treatment, and Followup

Approximately 18 months following the completion of initial therapy, the patient presented for routine follow-up care and was found to have elevated urine catecholamines. Workup revealed new diffuse skeletal MIBG uptake and marrow disease. Chemotherapy with cytoxan and topotecan produced a near complete response.

**Figure 1:**
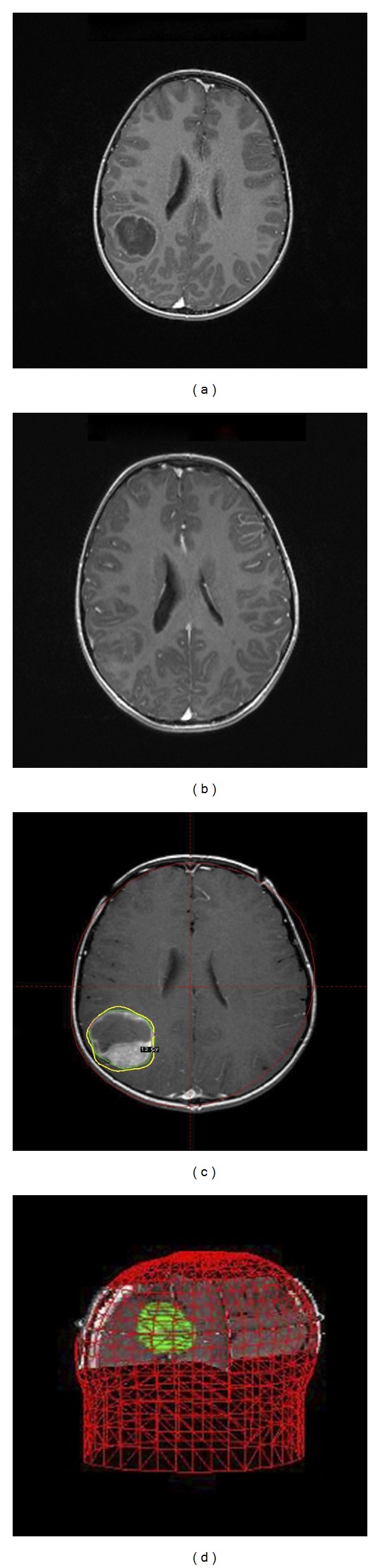
Neuroblastoma CNS metastasis treated with Gamma Knife stereotactic radiosurgery. (a) A T1-weighted brain magnetic resonance (MR) image from patient 1 following gadolinium administration demonstrates a 4.0 × 3.3 cm mass in the right parietal lobe. A thin rim of enhancement surrounds the lesion, which contains both cystic and solid components. (b) A T1-weighted brain MR image from patient 1 with gadolinium administration two months following stereotactic radiosurgery (SRS) reveals contraction of the lesion. A reduction in the enhancing components is also demonstrated, though proteinaceous components are still seen within the lesion. (c) and (d) A screenshot of the Leksell GammaPlan 4C treatment planning software is shown for patient 1. The right parietal lesion is targeted in this sequence. A 3D illustration confirms (d) minimal toxicity to surrounding structures.

One year following relapse, the patient presented to the Emergency Department with fever and severe headache. Decreased motor strength was noted in the left upper extremity. Brain magnetic resonance imaging (MRI) showed a 4.0 × 3.3 cm mass in the right parietal lobe with a small amount of surrounding edema and midline shift (Figure 1(a)). The patient was treated emergently with dexamethasone and subsequently underwent SRS (Figures 1(c) and 1(d)). Treatment was performed on a Leksell Gamma Knife Model 4C using Leksell GammaPlan 4C treatment planning software. A dose of 27.7 Gray (Gy), prescribed to the 47% isodose line, was administered to 18 targets. The 18 mm collimating helmet was used for 9 targets and the 14 mm collimating helmet was used for the remaining 9 targets. No individual collimator plugging was performed. The target volume was 29.2 cc and the volume of the prescribed 47% isodose line was 39.0 cc, yielding an excellent conformity index of 1.34. Target coverage by the prescribed isodose line was 100%. Follow-up imaging 2 and 4 months later revealed a stable decrease in the size of the mass (Figure 1(b)). The patient resumed chemotherapy and underwent additional craniospinal radiation with 21.6 Gy, the previously irradiated spinal portion receiving only 14.4 Gy. The patient also underwent total surgical resection of the intracranial mass three months later along with intra-Ommaya therapy. There was no evidence of recurrent CNS disease. The patient ultimately succumbed to disease 15 months after CNS relapse.

### 2.2. Case 2

#### 2.2.1. Initial Diagnosis and Treatment

A 2 1/2 -year-old male presented to our clinic with two months of fevers, weight loss, and lower extremity pain. On physical exam, an abdominal mass was palpable as well as a mass on the left frontal skull. Initial CT scan demonstrated a right suprarenal mass, and further workup revealed stage IV MYCN nonamplified neuroblastoma with elevated urinary catecholamines, bone marrow infiltration, and diffuse skeletal involvement, including the calvarium. The patient began treatment using the ANBLOOP1 protocol. Persistent marrow disease required additional cycles of chemotherapy to become negative, and the patient eventually underwent surgical resection of the primary tumor followed by HDC/SCR.

#### 2.2.2. Recurrence, Gamma Knife Treatment, and Followup

Bone marrow relapse occurred 1 month after SCR prior to local radiation. Isotretinoin was started. The patient received various alternative chemotherapy regimens including Zometa with Cytoxan, irinotecan with temozolomide, and lestaurtinib. Surveillance brain MRI performed 9 months after relapse showed a new predominantly solid mass in the left parietooccipital lobe measuring 1.8 × 1.0 cm with a small anterior cystic component (figure not shown). Cerebral stereotactic biopsy of the mass demonstrated neuroblastoma.

The patient was started on dexamethasone and subsequently treated with SRS. Treatment was accomplished on a Leksell Gamma Knife Model 4C using Leksell GammaPlan 4C treatment planning software. A dose of 14 Gy, prescribed to the 50% isodose line, was administered to 5 targets. The 18 mm collimating helmet was utilized. Follow-up imaging at 2 and 3 months showed a decrease in the size of the parietooccipital lesion, though head CT performed 4 months after SRS showed a slight increase in the size of the metastatic lesion with surrounding vasogenic edema. The patient ultimately succumbed to disease approximately 7.5 months following CNS relapse. 

## 3. Discussion

In the last half century, we have witnessed the extraordinary expansion of radiosurgical applications to a broad array of intracranial neoplasms, including both intra- and extra-axial lesions [13]. Primary CNS neuroblastoma has been previously treated with SRS by Oyama and colleagues, who documented reduction in the size of an intrasellar neuroblastoma treated via Gamma Knife radiotherapy (Table 1) [9]. Sakurada and colleagues also recorded a case of transformed neuroblastoma in the fourth ventricle and its temporary reduction in size with SRS [10]. Unger and colleagues yielded favorable results with combination endoscopic resection and radiosurgery in the largest reported esthesioneuroblastoma series [11]. SRS has not been previously applied to IMN.

Neuroblastoma is a complex systemic malignancy requiring multimodal therapy and multidisciplinary management. The current standard of care in neuroblastoma treatment is based on risk stratification into low-, intermediate-, or high-risk groups according to the COG classification system [3]. This scheme incorporates factors closely associated with outcome in neuroblastoma treatment: tumor histopathology, MYCN amplification, DNA ploidy, and the International Neuroblastoma Staging System (INSS) stage [2]. The mainstay of treatment for the high-risk group currently includes intensive chemotherapy, surgical resection, myeloablative consolidation therapy with autologous stem cell rescue, radiation therapy to the primary tumor site and persistent metastases, and biologic agents such as 13-*cis*-retinoic acid and anti-disialogangliosides [3]. We describe two cases of the application of SRS to high-risk, recurrent IMN. Both patients experienced reduction of tumor burden and transient stability of intracranial metastatic disease following treatment with Gamma Knife radiosurgery.

It is unclear whether the incidence of CNS recurrence is increasing. Most current chemotherapeutics do not penetrate the CNS, and there is concern that with modern increased survival rates the CNS may emerge as a sanctuary site [4]. It is estimated that five percent of cases present with CNS disease at initial diagnosis, though IMN may occur in up to 16% of patients with recurrent disease [14]. This further underscores the need for consideration of SRS as a viable treatment adjunct for IMN, depending on the clinical scenario. To date, enhancement of the efficacy of SRS by other treatment modalities in cases of IMN has not been addressed in the literature, though Croog and colleagues recently documented remission in a cohort of patients with recurrent IMN using conventional craniospinal irradiation paired with intra-Ommaya radioimmunotherapy [12]. With increased survival from more aggressive treatment protocols, SRS should be evaluated in the context of an expanded need in the near future for palliative and potentially curative treatment adjuncts for IMN. Furthermore, the advantage of delivering radiation in a single fraction with reductions in tumor burden comparable with conventional radiotherapy (CRT) must be exploited. Unfortunately, both patients in our report had widely metastatic disease and were considered incurable. SRS in both instances was a useful palliative tool that reduced tumor burden and provided symptomatic relief during the final stages of their illnesses. Future clinical studies may define optimal patient population attributes that may benefit most from this promising therapeutic intervention.

## Figures and Tables

**Table 1:** Comparison between modalities of radiation therapy for CNS neuroblastoma.

First author, year	No. of patients	Tumor pathology	Modality	Dosage	Time to recurrence/death
Oyama et al., 2005 [9]	1	Primary intrasellar neuroblastoma	GKSRS, CRT	15 Gy, 50 Gy	48 mo, 3 mo (NPALF)
Unger et al., 2005 [11]	14	Esthesioneuroblastoma	GKSRS	15–34 Gy	6–79 mo
Sakurada et al., 2007 [10]	1	Primary 4th ventricular neuroblastoma (transformed from neurocytoma)	GKSRS, WBRT	ND, 30 Gy	96 mo (PNAD)
Croog et al., 2010 [12]	29	Recurrent cerebral neuroblastoma	CSI CRT	1260–2160 cGy	1.5–63 mo

GKSRS: Gamma Knife Stereotactic Radiosurgery; GY: Gray; CRT: conventional radiation therapy; NPALF: no progression at last followup; WBRT: whole brain radiotherapy; ND: not documented; PNAD: progression noted at death; CSI: craniospinal irradiation; Cgy: centiGray.
